# Supplementary material for: Pyrimethamine and a potent analogue WCDD115 inhibit NRF2 by suppressing DHFR and one-carbon metabolism
Source: bioRxiv. 2025 Feb 17:2025.02.13.637433. Preprint. [Version 1] doi: 10.1101/2025.02.13.637433 (PMC11870417; doi:10.1101/2025.02.13.637433)
Supplement: Supplement 1 [file NIHPP2025.02.13.637433v1-supplement-1.pdf]

bioRxiv preprint doi: https://doi.org/10.1101/2025.02.13.637433; this version posted February 17, 2025. The copyright holder for this preprint (which was not certified by peer review) is the author/funder, who has granted bioRxiv a license to display the preprint in perpetuity. It is made available under aCC-BY 4.0 International license.

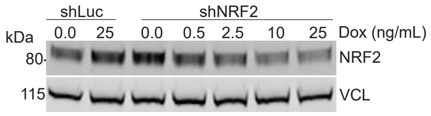

Supplemental Figure 1. Correlations between *NRF2* RNA abundance and NRF2 protein abundance. Representative Western blot image used to produce quantitative data shown in Figure 1H.

bioRxiv preprint doi: https://doi.org/10.1101/2025.02.13.637433; this version posted February 17, 2025. The copyright holder for this preprint (which was not certified by peer review) is the author/funder, who has granted bioRxiv a license to display the preprint in perpetuity. It is made available under aCC-BY 4.0 International license.

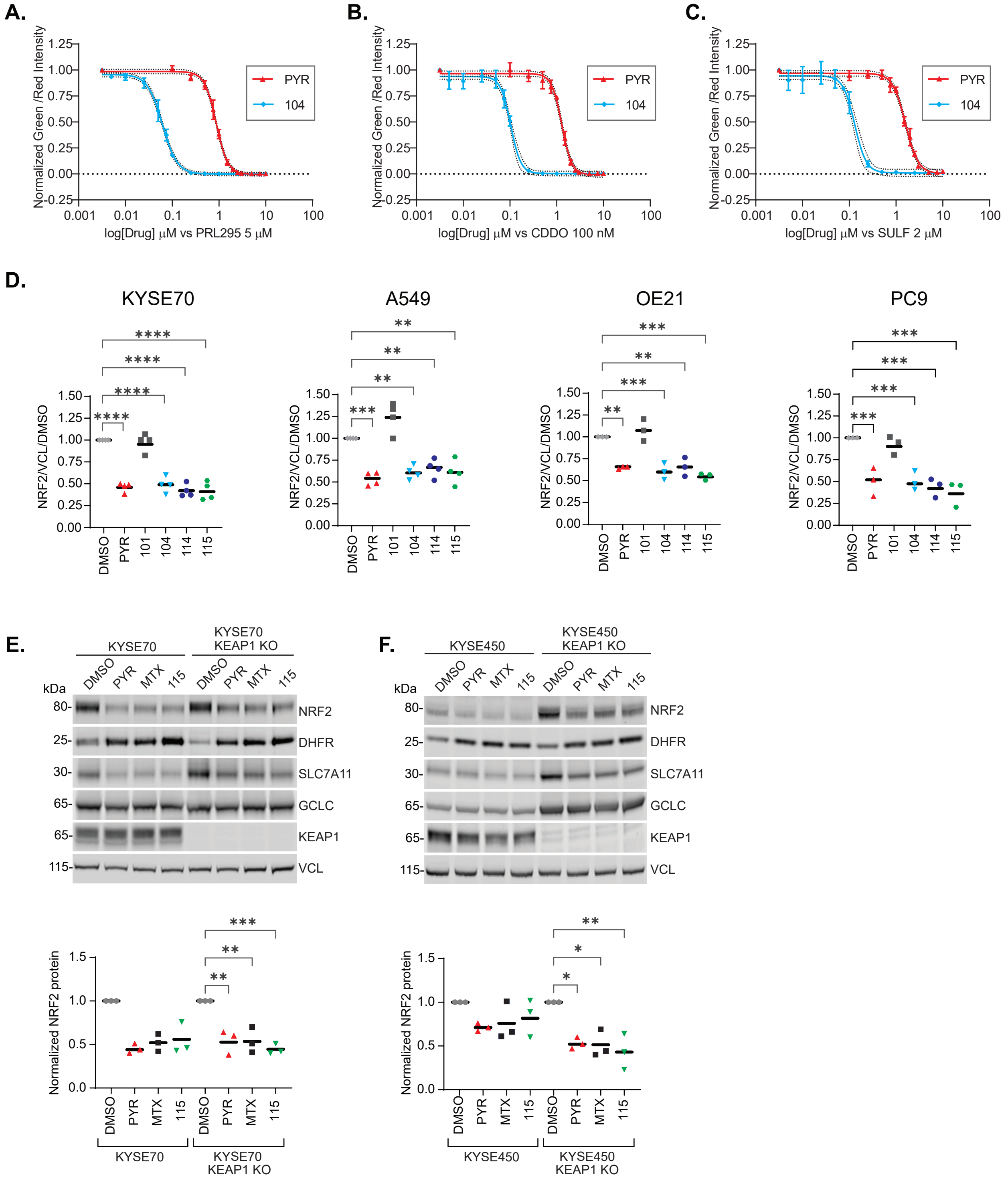

Figure S2

bioRxiv preprint doi: https://doi.org/10.1101/2025.02.13.637433; this version posted February 17, 2025. The copyright holder for this preprint (which was not certified by peer review) is the author/funder, who has granted bioRxiv a license to display the preprint in perpetuity. It is made available under aCC-BY 4.0 International license.

Supplemental Figure 2. KEAP1-independent suppression of NRF2 by PYR and its structural analogues. A-C. H1299 NQO1-eYFP cells were treated with increasing doses of PYR or WCDD104 in the presence of PRL295, CDDOme or 2 μM SULF. eYFP intensity was normalized to cell number (mCherry positive) before normalization to DMSO vehicle. **D.** Quantified data from Western blots shown in Figure 2E-H (**P < 0.05, ***P < 0.01, ****P < 0.001,) by one-way ANOVA (n=4 biological replicates per group). **E-F.** Western blot analysis of KYSE70 and KYSE450 cells and their KEAP1 KO derivatives after 48h treatment with 10μM PYR, 1μM 115, or 0.1μM MTX. Below, quantitative, normalized data are plotted for NRF2/VCL.

bioRxiv preprint doi: https://doi.org/10.1101/2025.02.13.637433; this version posted February 17, 2025. The copyright holder for this preprint (which was not certified by peer review) is the author/funder, who has granted bioRxiv a license to display the preprint in perpetuity. It is made available under aCC-BY 4.0 International license.

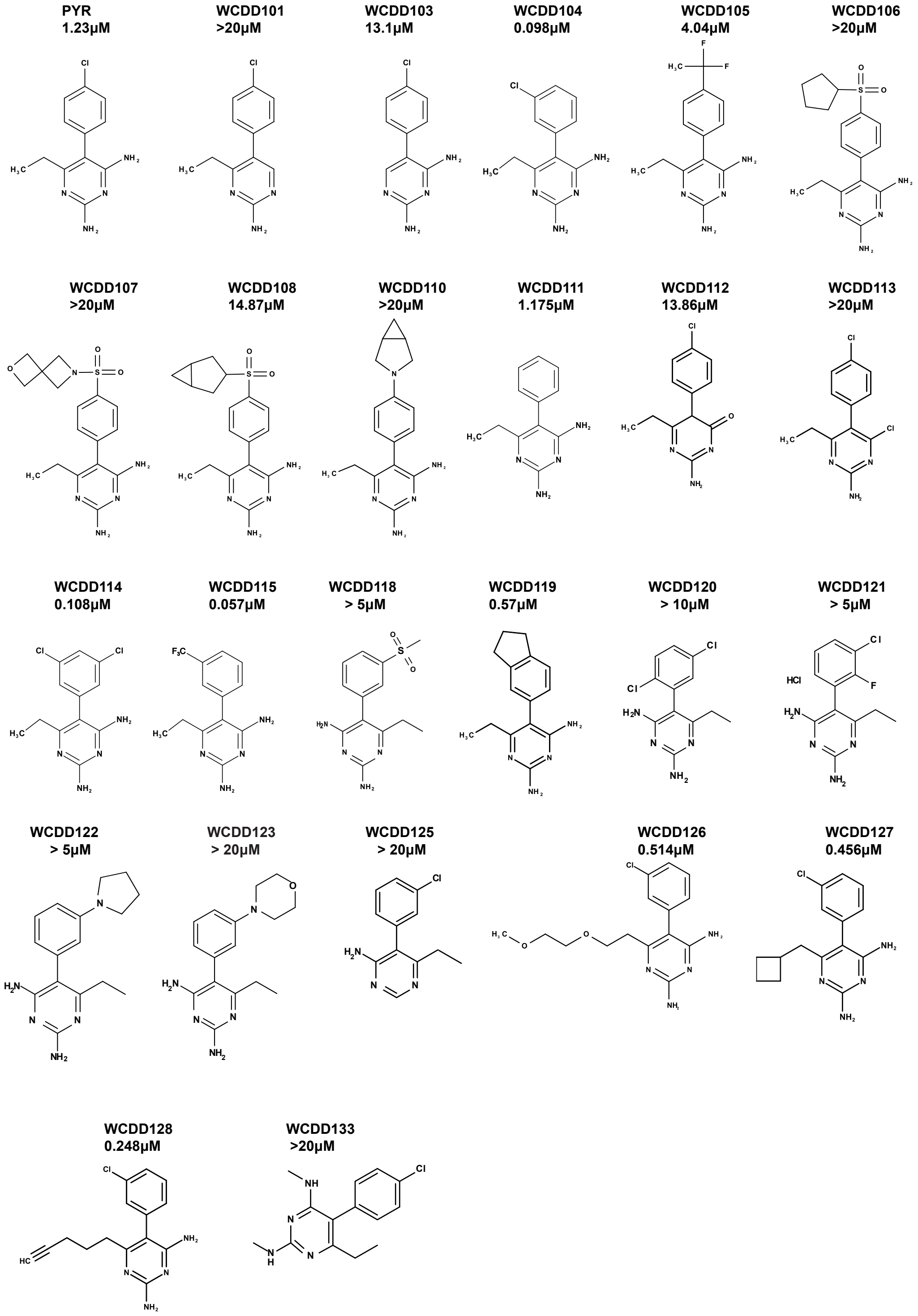

Supplemental Figure 3. Structures and NRF2 inhibition IC50 values of PYR analogues. IC50 values were defined by the H1299-NQO1-eYFP assay, taking the average IC50 values from SULF, PRL295 and CDDOme (see Table S2).

bioRxiv preprint doi: https://doi.org/10.1101/2025.02.13.637433; this version posted February 17, 2025. The copyright holder for this preprint (which was not certified by peer review) is the author/funder, who has granted bioRxiv a license to display the preprint in perpetuity. It is made available under aCC-BY 4.0 International license.

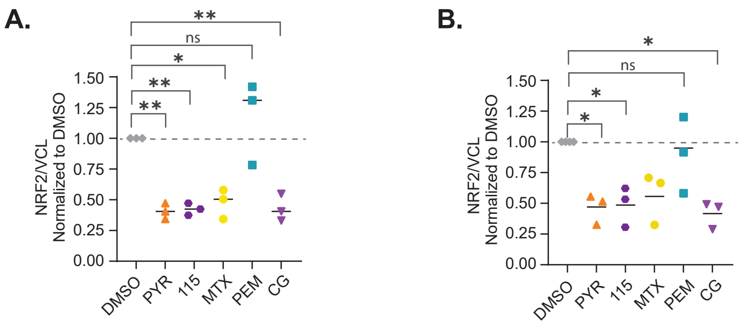

Supplemental Figure 4. Chemically distinct DHFR inhibitors suppress NRF2 signaling. A,B. Quantitative Western blot data associated with Figure 3D and 3E. Protein expression was normalized to VCL and then DMSO (*P < 0.05, **P < 0.01, ***P < 0.001 by one-way ANOVA across biological triplicate experiments).

bioRxiv preprint doi: https://doi.org/10.1101/2025.02.13.637433; this version posted February 17, 2025. The copyright holder for this preprint (which was not certified by peer review) is the author/funder, who has granted bioRxiv a license to display the preprint in perpetuity. It is made available under aCC-BY 4.0 International license.

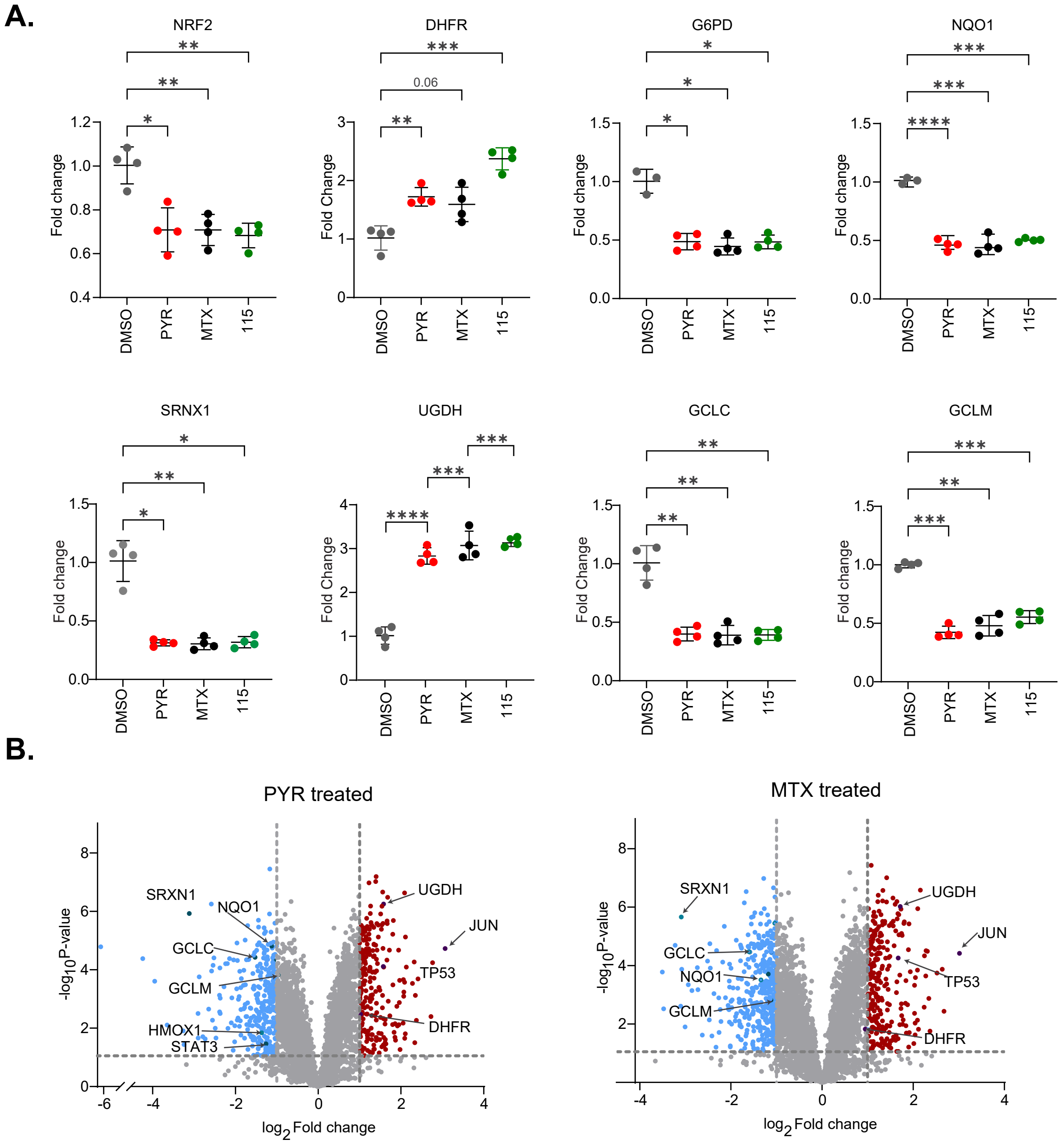

Supplemental Figure 5. **WCDD115 is an indirect inhibitor of NRF2**

A. OIS-PRM derived quantitation of the indicated proteins, derived from Figure 5A. Horizontal lines denote mean values, and asterisks indicate levels of statistical significance (*p<0.05; **p<0.01; ***p<0.001; ****p<0.0001). **B**. Volcano plot showing differentially expressed proteins in KYSE70 cells after 48h treatment with PYR or MTX.

bioRxiv preprint doi: https://doi.org/10.1101/2025.02.13.637433; this version posted February 17, 2025. The copyright holder for this preprint (which was not certified by peer review) is the author/funder, who has granted bioRxiv a license to display the preprint in perpetuity. It is made available under aCC-BY 4.0 International license.

Table S1. Cell line mutation status for NFE2L2 (NRF2) and KEAP1

Cell Line	NRF2 Alteration	KEAP1 Alteration
KYSE70	W24C	
A549		G333C
OE21	G81S, D318H	
PC9	copy number amplification	
H1299		
KYSE180	D77V	P278Q
H460		D236H
H2170		R336*
H1792		G462W
H2122		A170_R204del
KYSE450		

bioRxiv preprint doi: https://doi.org/10.1101/2025.02.13.637433; this version posted February 17, 2025. The copyright holder for this preprint (which was not certified by peer review) is the author/funder, who has granted bioRxiv a license to display the preprint in perpetuity. It is made available under aCC-BY 4.0 International license.

Table S2 Analog IC50 results in H1299-NQO1-eYFP cells vs activators

Compound	CDDO 100nM	PRL295 5μM	SULF 2μM	Avg IC50 μM	Modifed from
	IC50 mM + SEM				
PYR	1.245 ± 0.024	0.873 ± 0.032	1.591 ± 0.0448	1.23	N/A
WCDD101	>20	>20	>20	>20	PYR
WCDD103	16.86 ± 2.563	9.99 ± 0.0737	12.46 ± 0.798	13.10	PYR
WCDD104	0.103 ± 0.001	0.061 ± 0.003	0.129 ± 0.003	0.098	PYR
WCDD105	5.115 ± 0.074	3.743 ± 0.4988	3.279 ± 0.006	4.04	PYR
WCDD106	>20	>20	>20	>20	PYR
WCDD107	>20	>20	>20	>20	PYR
WCDD108	16.85 ± 0.208	15.21 ± 1.295	12.56 ± 0.1033	14.87	PYR
WCDD110	>20	>20	>20	>20	PYR
WCDD111	1.105 ± 0.016	1.511 ± 0.0329	0.910 ± 0.010	1.175	PYR
WCDD112	19.84 ± 0.338	10.83 ± 0.620	10.92 ± 0.0655	13.86	PYR
WCDD113	>20	>20	>20	>20	PYR
WCDD114	0.134 ± 0.014	0.079 ± 0.002	0.111 ± 0.009	0.108	WCDD104
WCDD115	0.052 ± 0.0001	0.060 ± 0.001	0.057 ± 0.003	0.057	WCDD104
WCDD118	>5	>5	>5	>5	WCDD104
WCDD119	0.614 ± 0.021	0.552 ± 0.003	0.557 ± 0.03	0.57	WCDD104
WCDD120	>10	>10	>10	>10	WCDD104
WCDD121	>5	>5	>5	>5	WCDD104
WCDD122	>5	>5	>5	>5	WCDD104
WCDD123	>20	>20	>20	>20	WCDD104
WCDD125	>20	>20	>20	>20	WCDD104
WCDD126	0.461 ± 0.017	0.683 ± 0.002	0.397 ± 0.011	0.514	WCDD104
WCDD127	0.469 ± 0.002	0.444 ± 0.002	0.454 ± 0.006	0.456	WCDD104
WCDD128	0.244 ± 0.006	0.334 ± 0.004	0.163 ± 0.002	0.248	WCDD104
WCDD133	>20	>20	>20	>20	WCDD104
